# Polymorphisms in *Plasmodium vivax* antifolate resistance markers in Afghanistan between 2007 and 2017

**DOI:** 10.1186/s12936-020-03319-0

**Published:** 2020-07-14

**Authors:** Kasama Rakmark, Ghulam R. Awab, Jureeporn Duanguppama, Usa Boonyuen, Arjen M. Dondorp, Mallika Imwong

**Affiliations:** 1 Department of Molecular Tropical Medicine and Genetics, Faculty of Tropical Medicine, Mahidol University, 420/6 Rajvithi Road, Bangkok, 10400 Thailand; 2 Nangarhar Medical Faculty, Ministry of Higher Education, Nangarhar University, Jalalabad, Afghanistan; 3 Centre for Tropical Medicine and Global Health, Nuffield Department of Medicine, University of Oxford, Oxford, UK; 4 Mahidol-Oxford Tropical Medicine Research Unit (MORU), Faculty of Tropical Medicine, Mahidol University, Bangkok, Thailand

**Keywords:** *Plasmodium vivax*, Antifolate resistance, Dihydrofolate reductase (DHFR) and dihydropteroate synthase (DHPS), Afghanistan

## Abstract

**Background:**

*Plasmodium vivax* is the predominant *Plasmodium* species in Afghanistan. National guidelines recommend the combination of chloroquine and primaquine (CQ-PQ) for radical treatment of *P. vivax* malaria. Artesunate in combination with the antifolates sulfadoxine-pyrimethamine (SP) has been first-line treatment for uncomplicated falciparum malaria until 2016. Although SP has been the recommended treatment for falciparum and not vivax malaria, exposure of the *P. vivax* parasite population to SP might still have been quite extensive because of community based management of malaria. The change in the *P. vivax* antifolate resistance markers between 2007 and 2017 were investigated.

**Methods:**

Dried blood spots were collected (n = 185) from confirmed *P. vivax* patients in five malaria-endemic areas of Afghanistan bordering Tajikistan, Turkmenistan and Pakistan, including Takhar, Faryab, Laghman, Nangarhar, and Kunar, in 2007, 2010 and 2017. Semi-nested PCR, RFLP and nucleotide sequencing were used to assess the pyrimethamine resistant related mutations in *P. vivax dihydrofolate reductase* (*pvdhfr* I13L, P33L, N50I, F57L, S58R, T61I, S93H, S117N, I173L) and the sulfonamide resistance related mutations in *P. vivax dihydropteroate synthase* (*pvdhps* A383G, A553G).

**Results:**

In the 185 samples genotyped for *pvdhfr* and *pvdhps* mutations*,* 11 distinct haplotypes were observed, which evolved over time. In 2007, wild type *pvdhfr* and *pvdhps* were the most frequent haplotype in all study sites (81%, 80/99). However, in 2017, the frequency of the wild-type was reduced to 36%, (21/58; *p *value ≤ 0.001), with an increase in frequency of the double mutant *pvdhfr* and *pvdhps* haplotype S58RS117N (21%, 12/58), and the single *pvdhfr* mutant haplotype S117N (14%, 8/58). Triple and quadruple mutations were not found. In addition, *pvdhfr* mutations at position N50I (7%, 13/185) and the novel mutation S93H (6%, 11/185) were observed. Based on in silico protein modelling and molecular docking, the *pvdhfr* N50I mutation is expected to affect only moderately pyrimethamine binding, whereas the S93H mutation does not.

**Conclusions:**

In the course of ten years, there has been a strong increase in the frequency pyrimethamine resistance related mutations in *pvdhfr* in the *P. vivax* population in Afghanistan, although triple and quadruple mutations conferring high grade resistance were not observed. This suggests relatively low drug pressure from SP on the *P. vivax* parasite population in the study areas. The impact of two newly identified mutations in the *pvdhfr* gene on pyrimethamine resistance needs further investigation.

## Background

With a sharp decline in falciparum malaria, *Plasmodium vivax* has become the prominent *Plasmodium* species in Afghanistan causing more than 95% of all malaria cases [1]. The recommended first-line treatment for vivax malaria in Afghanistan is chloroquine combined with primaquine for radical cure (CQ-PQ), whereas for uncomplicated falciparum malaria the combination of artesunate and sulfadoxine-pyrimethamine (AS-SP) has been first-line treatment from 2003 to 2016, replaced by artemether-lumefantrine since 2016. Although SP has been the recommended treatment for falciparum and not vivax malaria, exposure of the *P. vivax* parasite population to SP might still have been quite extensive because of community based management of malaria particularly symptom-based clinical or probable diagnosed malaria in past two decades [2, 3] and SP treatment of vivax malaria in parts of the private sector or through self-medication [3]. This might have increased antifolate resistance in *P. vivax* in Afghanistan.

Dihydrofolate reductase (DHFR) and dihydropteroate synthase (DHPS) are 2 essential enzymes in de novo folate synthesis pathway in *Plasmodium* and the drug targets of pyrimethamine, and sulfadoxine, respectively [4]. SP is considered a sub-optimal treatment for *P. vivax* infections, because this parasite is intrinsically less sensitive to sulfonamides and resistance to SP is rapidly acquired with extensive drug exposure [5]. Through clinical, epidemiological, molecular and biochemical studies it has been identified that SP resistance in *P. vivax* is conferred by specific point mutations in the *P. vivax* dihydrofolate reductase (*pvdhfr*) and dihydropteroate synthase (*pvdhps*) genes. In areas with extensive SP use, treatment failure of vivax malaria with SP rapidly evolved, and was associated with mutations in codons 57, 58, 61, 117, 173 of *pvdhfr* and in codons 382, 383, and 553 of *pvdhps* [6–10].

The frequencies of SP resistance related *pvdhf*r and *pvdhps* mutations have been extensively reported from various malaria-endemic areas, including Thailand [8, 11–14], Cambodia [15], Myanmar [16], Vietnam [17], Indonesia [18], Papua New Guinea [19], Madagascar [20] and India [21–24]. However, reports from Afghanistan and Middle East countries are scarce. Previous studies from Afghanistan [25], Iran [26, 27], Pakistan [28–31] showed a majority of *P. vivax* strains carried wild-type *pvdhf*r and *pvdhps*, and a low frequency in *pvdhfr* of the double mutation in codon S58R/ S117N and single mutation in codon S117N.

In this study, the frequency of SP resistance related point mutations in the *P. vivax pvdhfr* and *pvdhps* genes in Afghanistan over time between 2007 and 2017 were reported. In addition, in silico three-dimensional modelling and molecular docking of 2 newly identified *pvdhfr* mutationswere performed to predict their impact on pyrimethamine sensitivity.

## Methods

### Study sites and sample collection

Dried blood spots (n = 185) from patients with light microscopy confirmed *P. vivax* infection were collected from five malaria endemic provinces in Afghanistan. In 2007 samples were collected from Takhar (bordering Tajikistan), Faryab (bordering Turkmenistan), and Nangarhar (bordering Pakistan). In 2010, samples were collected from Kunar and in 2017 from Nangarhar and Laghman, all bordering Pakistan. Blood samples of 20–30 µl were collected onto filter paper at the moment of patient presentation before antimalarial treatment. Genomic DNA was extracted by using QIAmp® DNA Mini Kit (Qiagen, Hilden, Germany), according to the manufacturer’s instructions and purified DNA was stored at − 20 °C until further processing. Approval for this study was obtained from the Ethics Review Committee for Research in Human Subjects, Faculty of Tropical Medicine, Mahidol University, Thailand (EC approval number MUTM 2019-065-01).

In this study, the frequency of mixed infection of falciparum and vivax malaria was investigated by nested PCR detected 18S rRNA of *Plasmodium* spp. [32], only one sample was *P. vivax* and *P. falciparum* mixed infection (0.54%, 1/185). The low frequency of mixed infection of *P. vivax* and *P. falciparum* was explained by *P. falciparum* infected samples were excluded in the beginning.

### *Pvdhfr *and *pvdhps* amplification and genotyping

Amplification of *pvdhfr* by semi-nested Polymerase Chain Reaction was performed following established and published methods protocols [8, 23]. For amplification, the primary and secondary reaction volumes were 25 and 100 µl, respectively, including 1 µl of template genomic DNA added in the primary amplified reaction, and 3 µl of primary amplified product added to the second round of amplification. The reaction mixture contained a final concentration of 125 nM primers forward-reverse mixture, 10 mM Tris–HCL (pH 8.3), 2 mM MgCl2, 125 µM dNTP mixture, and 0.4 U Taq polymerase (Invitrogen, Carlsbad, CA) (Additional file 1: Table S1–S7). The DNA fragments from PCR amplification or Restriction Fragment Length Polymorphism (New England BioLabs Inc., Ipswich, MA) were identified by 3% metaphor agarose gel electrophoresis (Radnor Corporate Center Radnor, PA). The amplified PCR product of *pvdhfr* was purified for DNA sequencing using PCR purification kit, FavorPrep™ (Favorgen, Taiwan). The purified PCR products of *pvdhfr* were sequenced by Macrogen, Korea. Nucleotide and amino acid sequences of this gene were aligned and compared with the *P. vivax* reference sequence from the original *Sal1* strain (accession no. XM001615032), using BioEdit v7.2.5. For identification of established gene mutations associated with sulfonamide resistance, genotyping of *pvdhps* used PCR–RFLP methods, following established and published protocols [33]. The positive control of *P. vivax* is genomic DNA from *SalI* reference strain and characterized *P. vivax* isolates from the patient. To ensure the accuracy of the results, two positive controls and negative controls were added for quality control at every step of the procedure.

### In silico modelling of mutant PvDHFR

A computer simulated three-dimensional model of the PvDHFR protein structure and its putative interactions with pyrimethamine was used to predict the impact of *pvdhfr* mutations on pyrimethamine binding. The wild-type PvDHFR structure (PDB ID: 2BL9) was used as a template for mutant PvDHFR modelling. The model was constructed using SWISS-MODEL (https://swissmodel.expasy.org). The constructed model was verified by PROCHECK [34]. Then, the derived structures of the active sites of mutant PvDHFR were complexed with pyrimethamine and evaluated by AutoDock Vina [35]. The structural models were visualized by Discovery Studio Visualizer–Accelrys.

### Statistical analysis

The data of SNPs frequency related drug resistant genes were analysed by using MS Excel and SPSS v26.0. IBM Corp., Armonk, NY, USA. Pearson’s Chi-square test was used to compare proportions of haplotypes. A *P *value < 0.05 was considered statistically significant.

## Results

### *Pvdhfr* and *pvdhps* haplotypes

A total of 185 dry blood spots from patients presenting with vivax malaria were collected from the study sites. In the 185 genotyped samples, a total of 11 distinct *pvdhfr* and *pvdhps* haplotypes could be distinguished. Overall, the wild-type haplotype was the most frequent (64%, 119/185) (Table 1). Two other common haplotypes were a single mutant haplotype S117N (10%, 19/185) and a double mutant haplotype S58RS117N (9%, 16/185). Rare haplotypes included a mutation at codon position 383 of *pvdhps* in combination with *pvdhfr* single mutation at position 117; S117N/A383G (1%, 2/185) and a *pvdhfr* double mutations at 58 and 117; S58RS117N/A383G (1%, 1/185). The mutation A383G of *pvdhps* was detected in 8%, (3/37) of samples from Nangarhar in 2017.

**Table 1:** The Combination of *pvdhfr* and *pvdhps* mutations of *P. vivax* comparing by time and provinces

*Pvdhfr*									*Pvdhps*		Haplotypes	Faryab	Takhar	Nangarhar	Kunar	Nangarhar	Laghman	Total
I13L	P33L	N50I	F57L	S58R	T61M	S93H	S117N	I173L	A383G	A553G	*pvdhfr / pvdhps*	2007	2007	2007	2010	2017	2017	n = 185
												n = 30	n = 30	n = 39	n = 28	n = 37	n = 21	
I	P	N	F	S	T	S	S	I	A	A	WT / WT	23 (77%)	24 (80%)	33 (85%)	18 (64%)	12 (32%)	9 (43%)	119 (64%)
I	P	I	F	S	T	S	S	I	A	A	N50I / WT	1 (3%)				1 (3%)		3 (2%)
I	P	NI	F	S	T	S	S	I	A	A				1 (3%)				
I	P	N	F	R	T	S	S	I	A	A	S58R / WT						1 (5%)	3 (2%)
I	P	N	F	SR	T	S	S	I	A	A		1 (3%)		1 (3%)				
I	P	N	F	S	T	H	S	I	A	A	S93H / WT	2 (7%)	1 (3%)	1 (3%)	1 (4%)	3 (8%)		8 (4%)
I	P	N	F	S	T	S	N	I	A	A	S117N / WT		5 (17%)	1 (3%)	5 (18%)	5 (14%)	3 (14%)	19 (10%)
I	P	I	F	S	T	S	N	I	A	A	N50IS117N / WT	3 (10%)				5 (14%)	2 (10%)	10 (5%)
I	P	N	FL	SR	T	S	S	I	A	A	F57LS58R / WT			1 (3%)				1 (1%)
I	P	N	F	R	T	S	N	I	A	A	S58RS117N / WT			1 (3%)	3 (11%)	8 (22%)	4 (19%)	16 (9%)
I	P	N	F	S	T	H	N	I	A	A	S93HS117N / WT				1 (4%)		2 (10%)	3 (2%)
I	P	N	F	S	T	S	N	I	G	A	S117N / A383G					2 (5%)		2 (1%)
I	P	N	F	R	T	S	N	I	G	A	S58RS117N / A383G					1 (3%)		1 (1%)

*A* Ala, *F* Phe, *G* Gly, *H* His, *I* Ile, *L* Leu, *M* Met, *N* Asn, *P* Pro, *R* Arg, *S* Ser, *T* Thr, *FL* Phe/Leu (mixed infection), *NI* Asn/Ile (mixed infection), *SR* Ser/Arg (mixed infection)

A novel mutation at codon S93H (AGC to CAC) was identified comprising a single mutant haplotype S93H (4%, 8/185) and a double mutant haplotype S93HS117N (2%, 3/185) (Table 1). The N50I mutation (AAC to ATC) observed in this study had not been described from Afghanistan before. The mutation was part of the single N50I mutation haplotype present in 2% (3/185) of samples. While double mutant haplotype N50IS117N was found in 5% (10/185) of samples. Haplotypes with triple or quadruple mutations, which are associated with high grade antifolate resistance, were not observed in this study.

### Temporal changes in *pvdhfr* and *pvdhps* haplotypes between 2007 and 2017

**Fig. 1:**
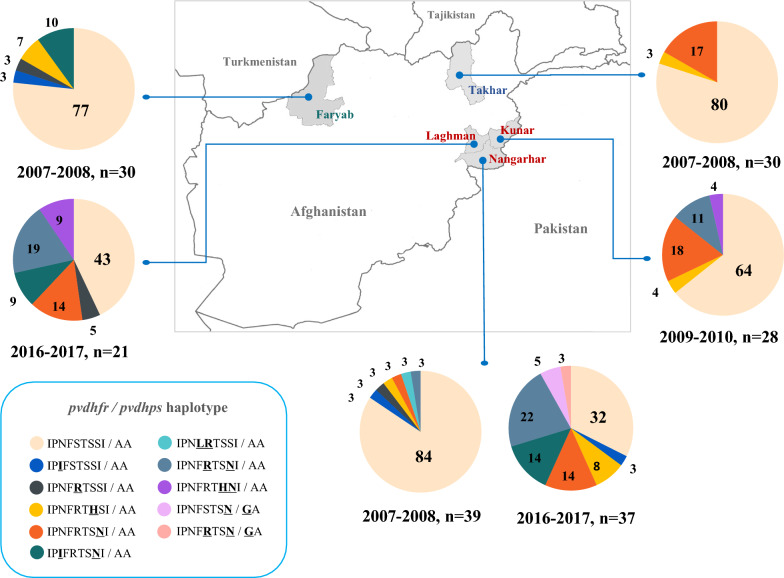
Frequencies of mutations (%) in *pvdhfr* and *pvdhps* in five border provinces of Afghanistan. Nine non-synonymous mutant codons of *pvdhfr;* I13L, P33L, N50I, F57L, S58R, T61I, S93H, S117N, I173L and 2 codon non-synonymous mutant codons of *pvdhps*; A383G, A553G were observed

**Fig. 2:**
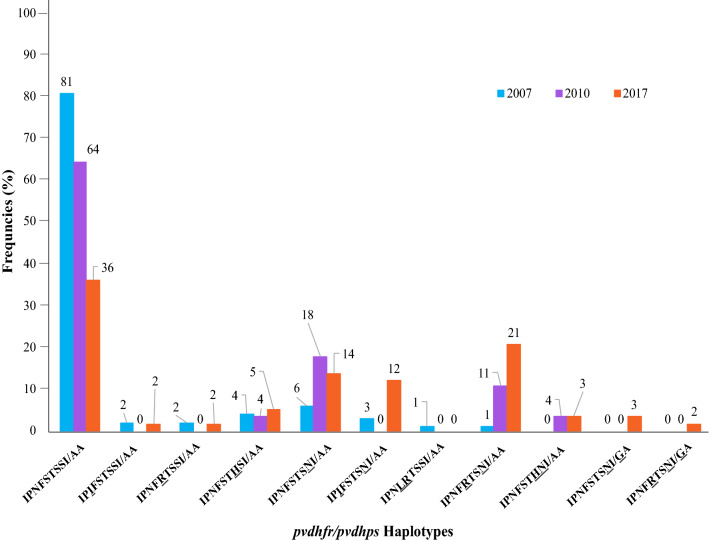
Comparison of *pvdhfr/pvdhps* haplotype frequncies (%) between year 2007 to 2017

In 2007, the wild type *pvdhfr* and *pvdhps* haplotype was most frequent (81%, 80/99). Other haplotypes were rare, including the single mutant haplotype S117N in 6%, (6/99), and the double mutant haplotype N50IS117N in 3% (3/99) of samples. Also in the year 2010 the wild type *pvdhfr* and *pvdhps* haplotype was most frequent (64%, 18/28). The single mutant haplotype S117N (18%, 5/28) and double mutant haplotype S58RS117N (11%, 3/28) were also observed. In contrast in 2017, the frequency of wild-type *pvdhfr* and *pvdhps* haplotype was compared to 2007 significantly reduced to 36% (21/58) of samples (*p *value ≤ 0.001). In 2017, the frequencies of the single mutant haplotype S117N (14%, 8/58, *p *value ≤ 0.001) and double mutant haplotype S58RS117N had increased significantly (21%, 12/58, *p *value ≤ 0.001) (Table 1) (Figs. 1, 2).

### In silico modelling of novel *pvdhfr* mutations

**Fig. 3:**
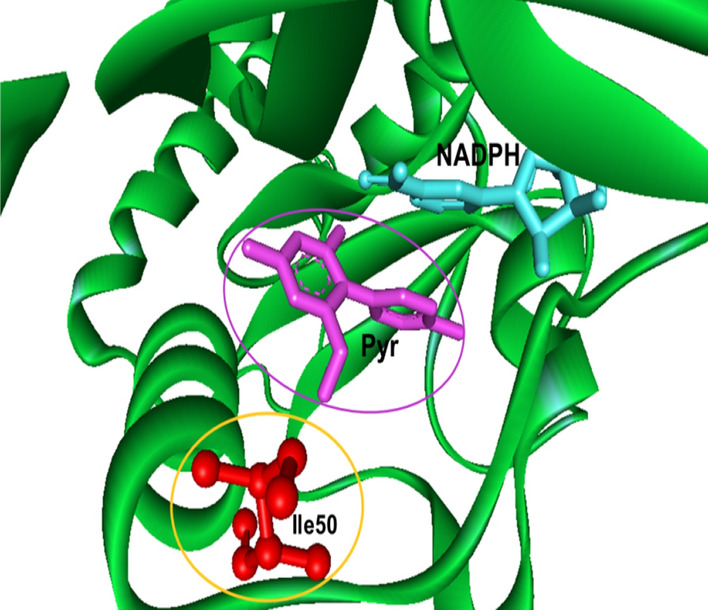
Structural model of PvDHFR N50I mutation complexed with pyrimethamine. A mutation at position 50 (in yellow circle) is located within the binding pocket of pyrimrthamine (in purple circle)

**Fig. 4:**
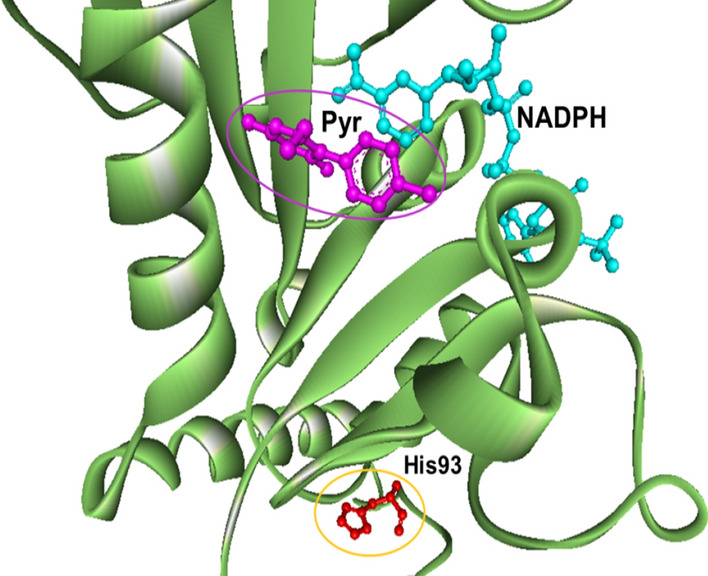
Structural model of PvDHFR S93H mutation complexed with pyrimethamine. A mutation at position 93 (in yellow circle) is located far away from the binding pocket of pyrimrthamine (in purple circle). This mutation does not have any effect on pyrimethamine binding

**Fig. 5:**
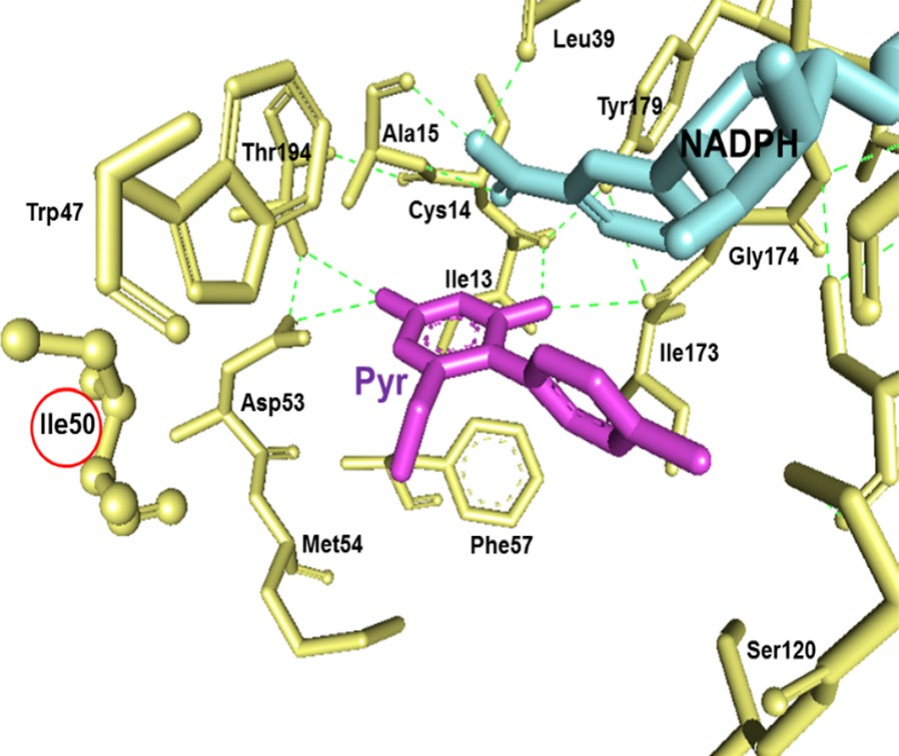
Molecular interactions of pyrimethamine and PvDHFR. The binding analysis showed that mutation at position 50 (in red circle) is located approximately 5°A from pyrimethamine (purple) but not directly involved in the binding

Since the two nonsynonymous *pvdhfr* mutation at position N50I, AAC (Asn) to ATC (Ile), and S93H, AGC (Ser) to CAC (His) had not been observed earlier in Afghanistan, the corresponding three-dimensional structures of the mutated proteins and the impact on pyrimethamine binding were modelled and predicted. The mutation N50I is located within the binding pocket, approximately 5 Å from the pyrimethamine molecule but does not directly interact with the drug. The mutation S93H is placed on the other side of the protein molecule, far away from the binding pocket (Figs. 3, 4, 5). Replacement of Asn with Ile at position 50 disrupts the hydrogen bonds between Asn and water molecules. The N50I mutation also causes a conformation rearrangement of the helix (residues 50–63), disturbing the favorable interactions between the enzyme and inhibitor. The model predicted that the *pvdhfr* N50I mutation would affect only moderately pyrimethamine binding, whereas the S93H mutation would not affect binding.

## Discussion

The combination of AS-SP has been used as first-line treatment for uncomplicated.

*P. falciparum* in Afghanistan since 2003 to 2016, and SP has been widely available in private sectors for a long time. Since an episode of falciparum malaria triggers relapse infection with *P. vivax* a few weeks later, this provides a window of selection for resistant parasites to outgrow sensitive parasite still killed by the remaining SP drug levels. Drug pressure from SP on the *P. vivax* parasite population over the past decade is thus likely, which might cause gene mutations in *pvdhfr* and *pvdhps* causing reduced SP sensitivity. Indeed, in this study, the frequency of the double mutant *pvdhfr* haplotype at S58RS117N and single mutant haplotype at S117N had increased significantly over the course of ten years since 2007 were observed. However, the triple and quadruple haplotype mutations associated with higher level of antifolate resistance were not found, suggesting only low or moderate drug pressure from SP on the *P. vivax* parasite populations in the study areas.

These findings agree with previously published reports showing low prevalence (less than 5%) of triple and quadruple mutant *pvdhfr* and *pvdhps* haplotypes in *P. vivax* parasite populations from Iran, Pakistan and India (Table 2) [21, 22, 26–31, 36–39]. In the wider study region, it was observed that the pyrimethamine resistance marker at position 117 in *pvdhfr* arose first and was followed by the subsequent mutation in positions 117 and 58, conferring increasing levels of resistance as report earlier [23, 26–31].

**Table 2:** Published molecular surveys of pyrimethamine and sulphadoxine markers in south-western Asia

Year of sampling	Countries	States/ provinces	*Pvdhfr*									*Pvdhps*		Haplotypes	Frequencies	Prevalence	References
			I13L	P33L	N50I	F57L	S58R	T61M	S93H	S117N	I173L	A383G	A553G	*pvdhfr / pvdhps*			
2008	Afghanistan	Herat	I	P	–	F	S	T	–	S	I	A	A	WT / WT	92% (79/85)		
			I	P	–	F	S	T	–	N	I	A	A	S117N / WT	5% (4/85)		
			I	P	–	F	R	T	–	N	I	A	A	S58RS117N / WT	1% (1/85)		[25]
			I	P	–	F	R	T	–	N	I	G	A	S58RS117N / A383G	1% (1/85)		
		Nangarhar	I	P	–	F	S	T	–	S	I	A	A	WT / WT	79% (68/86)		
			I	P	–	F	S	T	–	N	I	A	A	S117N / WT	15% (13/86)		
			I	P	–	F	R	T	–	N	I	A	A	S58RS117N / WT	6% (5/86)		
2007–2008	Afghanistan	Faryab	I	P	N	F	S	T	S	S	I	A	A	WT / WT	77% (23/30)		This study
			I	P	I	F	S	T	S	S	I	A	A	N50I / WT	3% (1/30)		
			I	P	N	F	R	T	S	S	I	A	A	S58R / WT	3% (1/30)		
			I	P	N	F	S	T	H	S	I	A	A	S93H / WT	7% (2/30)		
			I	P	I	F	S	T	S	N	I	A	A	N50IS117N / WT	10% (3/30)		
		Takhar	I	P	N	F	S	T	S	S	I	A	A	WT / WT	80% (24/30)		
			I	P	N	F	S	T	H	S	I	A	A	S93H / WT	3% (1/30)		
			I	P	I	F	S	T	S	N	I	A	A	S117N / WT	17% (5/30)		
		Nangarhar	I	P	N	F	S	T	S	S	I	A	A	WT / WT	85% (33/39)		
			I	P	I	F	S	T	S	S	I	A	A	N50I / WT	3% (1/39)		
			I	P	N	F	R	T	S	S	I	A	A	S58R / WT	3% (1/39)		
			I	P	N	F	S	T	H	S	I	A	A	S93H / WT	3% (1/39)		
			I	P	N	F	S	T	S	N	I	A	A	S117N / WT	3% (1/39)		
			I	P	N	L	R	T	S	S	I	A	A	F57LS58R / WT	3% (1/39)		
			I	P	N	F	R	T	S	N	I	A	A	S58RS117N / WT	3% (1/39)		
2009–2010	Afghanistan	Kunar	I	P	N	F	S	T	S	S	I	A	A	WT / WT	64% (18/28)		
			I	P	N	F	S	T	H	S	I	A	A	S93H / WT	4% (1/28)		
			I	P	N	F	S	T	S	N	I	A	A	S117N / WT	18% (5/28)		
			I	P	N	F	R	T	S	N	I	A	A	S58RS117N / WT	11% (3/28)		
			I	P	N	F	S	T	H	N	I	A	A	S93HS117N / WT	4% (1/28)		
2017	Afghanistan	Nangarhar	I	P	N	F	S	T	S	S	I	A	A	WT / WT	32% (12/37)		
			I	P	I	F	S	T	S	S	I	A	A	N50I / WT	3% (1/37)		
			I	P	N	F	S	T	H	S	I	A	A	S93H / WT	8% (3/37)		
			I	P	N	F	S	T	S	N	I	A	A	S117N / WT	14% (5/37)		
			I	P	I	F	S	T	S	N	I	A	A	N50IS117N / WT	14% (5/37)		
			I	P	N	F	R	T	S	N	I	A	A	S58RS117N / WT	22% (8/37)		
			I	P	N	F	S	T	S	N	I	G	A	S117N / A383G	5% (2/37)		
			I	P	N	F	R	T	S	N	I	G	A	S58RS117N / A383G	3% (1/37)		
		Lagman	I	P	N	F	S	T	S	S	I	A	A	WT / WT	43% (9/21)		
			I	P	N	F	R	T	S	S	I	A	A	S58R / WT	5% (1/21)		
			I	P	N	F	S	T	S	N	I	A	A	S117N / WT	14% (3/21)		
			I	P	I	F	S	T	S	N	I	A	A	N50IS117N / WT	10% (2/21)		
			I	P	N	F	R	T	S	N	I	A	A	S58RS117N / WT	19% (4/21)		
			I	P	N	F	S	T	H	N	I	A	A	S93HS117N / WT	10% (2/21)		
2007	Pakistan	Bannu	I	P	–	F	S	T	–	S	I	A	A	WT / WT		7%	[28]
			I	P	–	F	S	T	–	N	I	A	A	S117N / WT		75%	
			I	P	–	L	R	T	–	S	I	A	A	F57LS58R / WT		2%	
			I	P	–	F	R	T	–	N	I	A	A	S58RS117N / WT		16%	
2008	Pakistan	FATA	I	P	–	F	S	T	–	S	I	A	A	WT / WT	44% (72/164)		[30]
			I	P	–	F	R	T	–	S	I	A	A	S58R / WT	1% (3/164)		
			I	P	–	F	S	T	–	N	I	A	A	S117N / WT	40% (65/164)		
			I	P	–	F	S	T	–	S	I	G	A	WT / A383G	1% (1/164)		
			I	P	–	F	R	T	–	N	I	A	A	S58RS117N / WT	13% (22/164)		
			I	P	–	F	S	T	–	N	I	G	A	S117N / A383G	1% (1/164)		
2011	Pakistan	Baluchistan, Islamabad, Khyber Pakhtunkhwa, Punjab, Sindh,	I	P	–	F	S	T	–	S	I	A	A	WT / WT	44% (143/322)		
			I	P	–	F	R	T	–	S	I	A	A	S58R / WT	1% (3/322)		
			I	P	–	F	S	T	–	N	I	A	A	S117N / WT	30% (95/322)		
			I	P	–	F	S	T	–	S	I	G	A	WT / A383G	0.3% (1/322)		
			I	P	–	F	S	T	–	S	I	A	G	WT / A553G	0.3% (1/322)		
			I	P	–	L	R	T	–	S	I	A	A	F57LS58R / WT	1.6% (5/322)		[31]
			I	P	–	F	R	T	–	N	I	A	A	S58RS117N / WT	21% (69/322)		
			I	P	–	F	S	T	–	N	I	G	A	S117N / A383G	0.6% (2/322)		
			I	P	–	F	R	T	–	N	I	G	A	S58RS117N / A383G	0.6% (2/322)		
			I	P	–	F	R	T	–	N	I	G	G	S58RS117N / A383GA553G	0.3% (1/322)		
2008–2009	Pakistan	Karachi, Sindh, Baluchistan,	I	P	–	F	S	T	–	S	I	A	A	WT / WT	40% (51/131)		
			I	P	–	F	R	T	–	S	I	A	A	S58R / WT	2% (2/131)		
			I	P	–	F	S	T	–	N	I	A	A	S117N / WT	18% (24/131)		
			I	P	–	F	S	T	–	S	I	G	A	WT / A383G	7% (9/131)		
			I	P	–	L	R	T	–	S	I	A	A	F57LS58R / WT	2% (3/131)		
			I	P	–	F	R	T	–	N	I	A	A	S58RS117N / WT	25% (33/131)		[29]
			I	P	–	F	R	T	–	S	I	G	A	S58R / A383G	1% (1/131)		
			I	P	–	F	S	T	–	N	I	G	A	S117N / A383G	2% (3/131)		
			I	P	–	F	R	T	–	N	I	G	A	S58RS117N / A383G	3% (4/131)		
			I	P	–	F	R	T	–	N	I	G	G	S58RS117N / A383GA553G	1% (1/131)		
2001–2006	Iran	Sistan and Baluchistan	I	P	–	F	S	T	–	S	I	A	A	WT / WT	59% (85/143)		
			I	P	–	L	S	T	–	S	I	A	A	F57L / WT	1% (1/143)		
			I	P	–	F	R	T	–	S	I	A	A	S58R / WT	9% (13/143)		
			I	P	–	F	S	T	–	N	I	A	A	S117N / WT	18% (26/143)		
			I	P	–	L	R	T	–	S	I	A	A	F57LS58R / WT	1% (1/143)		
			I	P	–	F	R	T	–	N	I	A	A	S58RS117N / WT	12% (17/141)		[27]
		Ardebil	I	P	–	F	S	T	–	S	I	A	A	WT / WT	85% (39/46)		
			I	P	–	F	R	T	–	S	I	A	A	S58R / WT	2% (1/46)		
			I	P	–	F	S	T	–	N	I	A	A	S117N / WT	11% (5/46)		
			I	P	–	F	R	T	–	N	I	A	A	S58RS117N / WT	2% (1/46)		
2010–2015	Iran	Hormozgan	I	P	–	F	S	T	–	S	I	A	A	WT / WT	73% (58/80)		
			I	P	–	F	S	T	–	N	I	A	A	S117N / WT	11% (9/80)		
			I	P	–	F	R	T	–	N	I	A	A	S58RS117N / WT	8% (6/80)		[26]
			I	P	–	F	R	T	–	S	I	G	A	S58R / A383G	6% (5/80)		
			I	P	–	L	R	T	–	N	I	A	A	F57LS58RS117N / WT	3% (2/80)		
2003–2004	India	India	I	P	–	F	S	T	–	S	I	A	A	WT / WT		51%	
			I	P	–	F	S	T	–	N	I	A	A	S117N / WT		1%	[38]
			I	P	–	F	R	T	–	N	I	A	A	S58RS117N / WT		48%	
2011–2012	India	Aligarh	I	P	–	F	S	T	S	S	I	A	A	WT / WT	61% (28/46)		
			I	P	–	F	S	T	H	S	I	A	A	S93H / WT	7% (3/46)		[39]
			I	P	–	F	S	T	S	N	I	A	A	S117N / WT	7% (3/46)		
			I	P	–	F	R	T	S	N	I	A	A	S58RS117N / WT	26% (12/46)		
		Kolkata	I	P	–	F	S	T	–	S	I	A	A	WT / WT		35%	
			I	P	–	F	R	T	–	S	I	A	A	S58R / WT		3%	
			I	P	–	F	S	T	–	S	I	G	A	WT / A383G		24%	
			I	P	–	F	R	T	–	N	I	A	A	S58RS117N / WT		6%	[37]
			I	P	–	F	S	T	–	S	I	G	G	WT / A383GA553G		1%	
			I	P	–	F	R	T	–	N	I	G	A	S58RS117N / A383G		30%	
			I	P	–	F	R	T	–	N	I	G	G	S58RS117N / A383GA553G		1%	
2013–2014	India	KMC	I	P	–	F	S	T	–	S	I	A	A	WT / WT		37%	
			I	P	–	F	R	T	–	S	I	A	A	S58R / WT		6%	[36]
			I	P	–	F	R	T	–	N	I	A	A	S58RS117N / WT		54%	
			I	P	–	L	R	T	–	N	I	A	A	F57LS58RS117N / WT		4%	
		Purulia	I	P	–	F	S	T	–	S	I	A	A	WT / WT		4%	
			I	P	–	F	R	T	–	S	I	A	A	S58R / WT		15%	
			I	P	–	F	S	T	–	N	I	A	A	S117N / WT		9%	
			I	P	–	F	R	T	–	N	I	A	A	S58RS117N / WT		8%	
2014–2015	India	Mangaluru	I	P	–	F	R	T	–	N	I	A	A	S58RS117N / WT	100% (25/25)		[21]

Antifolate drugs inhibit two essential enzymes in the folate biosynthesis pathway, necessary for DNA precursor synthesis. Sulfadoxine inhibits DHPS while pyrimethamine inhibits DHFR, which results in parasite death [4]. In *P. falciparum*, the intensive use of SP as first line treatment in south-east Asia since 1970s resulted in rapid acquisition of mutations in *pfdhfr* and *pfdhps*, causing amino acid changes in the enzymes at positions in the binding pockets for pyrimethamine and sulfadoxine, respectively, causing SP resistance [7, 8]. The antifolate resistant mechanism in *P. vivax*, with incremental resistance acquired by the accumulation of single point mutations in *pvdhfr* and *pvdhps*, is thought to be similar to the antifolate resistance mechanism in *P. falciparum.* In *P. vivax,* the sequence of non-synonymous mutations in *pvdhfr* is in codons 13, 58, 117, 173, equivalent to the orthologous *pfdhfr* positions 16, 59, 108 and 164 in *P. falciparum.* In *P. vivax*, biochemical and protein functional studies showed that pyrimethamine effectively inhibits wild-type PvDHFR, but were approximately 60 to  > 4000 times less active against mutant enzymes. Double mutant S58R and S117N PvDHFR was 10–25 fold less inhibited by pyrimethamine than the S117N mutant [10]. This relates to the steric hindering of the pyrimethamine binding pocket caused by the S117N mutation in the PvDHFR enzyme [9, 10].The studies also showed that a change in the effect of pyrimethamine on the enzyme kinetic properties of mutant PvDHFR is closely related with a change in sensitivity to pyrimethamine [9, 10].

The current study identified a novel nonsynonymous mutation S93H in *pvdhfr* and for Afghanistan newly observed N50I mutation in *pvdhfr*. The N50I mutation corresponds to the orthologous mutation N51I in *P. falciparum*. The *pvdhfr* N50I mutation was previously observed in low frequency in Pakistan [29]. Previous study shown that mutant N50I PvDHFR expressed in yeast confers an increase in pyrimethamine IC_50_ compared to wild type PvDHFR, whereas, the combination of mutation N50I and S117N conferred a-57 fold increase in IC_50_ compared to wild type [40]. To assess the interaction of these mutations on pyrimethamine binding, homology modelling and molecular docking *in-silico* were performed. This showed that the *pvdhfr* N50I mutation interrupted moderately the binding of pyrimethamine. In contrast, the *pvdhfr* S93H mutation, changing amino acids from AGC (Ser) to CAC (His) located away from the binding pocket of pyrimethamine, was predicted not to interrupt pyrimethamine binding. However, these in silico results will need confirmation in further in vitro or in vivo drug sensitivity studies.

An additional marker for SP resistance in *P. falciparum* is increased copies number of GTP cyclohydrolase I gene (*pfgch1).* GTP cyclohydrolase is another important enzyme in folate biosynthesis, and an increased copy number might compensate for the fitness loss associated with triple and quadruple mutations in *pfdhfr* and *pfdhps* [41, 42]. So far, there is limited number of reports on gene amplification of *pvgch1*, therefore still no finding of the association of mutations in *pvdhfr/pvdhps* and *pvgch1* copy number variation. Whether copy number variation (CNV) in the *P. vivax* orthologous gene *pvgch1* is important in antifolate resistance needs further study. *Pvgch1* CNV was not assessed in the current study, but less likely to be present because of the absence of triple- or quadruple mutations *in pvdhfr* in our samples.

## Conclusions

This study shows that although wild-type *pvdhfr* and *pvdhps* haplotypes have become less frequent in the Afghanistan *P. vivax* parasite population between 2007 and 2017, but only double and single nonsynonymous mutations in *pvdhfr* were observed, which would confer only moderate SP resistance. This suggests relatively low drug pressure from SP on the *P. vivax* parasite population in the study areas. Of the two novel *pvdhfr* mutations identified in Afghanistan, only the N50I was predicted to moderately affect pyrimethamine binding.

## Supplementary information

**Additional file 1.** Details of PCR and RFLP conditions.

## Data Availability

The dataset generated during the current study are available from corresponding author on reasonable request.

## Abbreviations

*pvdhfr*: *Plasmodium vivax dihydrofolate reductase*
*pvdhps*: *P. vivax dihydropteroate synthase*
SNPs: Single nucleotide polymorphisms
CQ-PQ: Chloroquine and primaquine
SP: Artesunate and sulfadoxine-pyrimethamine
