# Supplementary material for: Polymorphisms in Plasmodium vivax antifolate resistance markers in Afghanistan between 2007 and 2017
Source: Malar J. 2020 Jul 14;19:251. doi: 10.1186/s12936-020-03319-0 (PMC7362531; doi:10.1186/s12936-020-03319-0)
Supplement: Supplementary file 1 — Additional file 1. Details of PCR and RFLP conditions. [file 12936_2020_3319_MOESM1_ESM.docx]

**Supplementary information**

Table S1 Primer sequences for detection *of* *pvdhfr* [8]

| **Codon** |  | **Primer** | **Sequence 5’-3’** | **Product size** |
| --- | --- | --- | --- | --- |
| 13, 33, 50, 57, 58,61, 93, 117, 173 | Nested 1 | VDT_OF | ATGGAGGACCTTTCAGATGTATTTGACATT | 1876 bp |
|  |  | VDT_OR | GGCGGCCATCTCCATGGTTATTTTATCGTG |  |
|  | Nested 2 | VDT_OF | ATGGAGGACCTTTCAGATGTATTTGACATT | 611 bp |
|  |  | VDF_NR | TCACACGGGTAGGCGCCGTTGATCCTCGTG |  |

Table S2 Primer sequences for detection *of* *pvdhps* [33]

| **Codon** | **Primer** | **Sequence 5’-3’** | **Product size** |
| --- | --- | --- | --- |
| 383, 553 | VDHPS-OF | ATTCCAGAGTATAAGCACAGCACATTTGAG | 1354 bp |
|  | VDHPS-OR | CTAAGGTTGATGTATAATTGTGAGCACATC |  |
| 383 | VDHPS-NF | AATGGCAAGTGATGGGGCGAGCGTGATTGA | 705 bp |
|  | VDHPS-NR | CAGTCTGCACTCCCCGATGGCCGCGCCACC |  |
| 553 | VDHPS-553OF | TTCTCTTTGATGTCGGCCTGGGGTTGGCCA | 171 |
|  | VDHPS-NR | CAGTCTGCACTCCCCGATGGCCGCGCCACC |  |

Table S3**.** Master mix Preparation of *pvdhfr* nested I PCR reaction [8]

| **Reagent** | **Final conc.** | **Volume** |
| --- | --- | --- |
| 10X PCR Buffer  50 mM MgCl2  5 mM dNTPs  2.5 µM Primers (mix Forward & Reverse)  Taq DNA Polymerase  H2O | 1X  2 mM  125 µM  125 nM  0.4 U | 2 µl  0.8 µl  0.5 µl  1 µl  0.08 µl  15.7 µl |
| Total |  | 25 µl |
| Template DNA | 1. µl |  |

Table S4. Master mix Preparation of *pvdhfr* nested II PCR reaction [8]

| **Reagent** | **Final conc.** | **VDT_OF/**  **VDF_NR** |
| --- | --- | --- |
| 10X PCR Buffer | 1X | 9 µl |
| 50 mM MgCl2 | 2 mM | 3.6 µl |
| 5 mM dNTPs | 125 µM | 2.25 µl |
| 2.5 µM Primers | 250 nM | 9 µl |
| (mix Forward & Reverse) |  |  |
| Taq Polymerase | 0.6 U |  |
|  | 1.8 U | 0.36 µl |
| H2O |  | 65.8 µl |
| Total |  | 100 µl |
| Template DNA from Nested 1 |  | 3 µl |

Table S5. Master mix Preparation of *pvdhps* nested I PCR reaction [33]

| **Reagent** | **Final conc.** | **Volume** |
| --- | --- | --- |
| 10X PCR Buffer  50 mM MgCl2  5 mM dNTPs  2.5 µM Primers (mix Forward & Reverse)  Taq DNA Polymerase  H2O | 1X  3 mM  125 µM  250 nM  0.4 U | 2 µl  1.2 µl  0.5 µl  2 µl  0.08 µl  14.3 µl |
| Total |  | 20 µl |
| Template DNA | 1 µl |  |

Table S6. Master mix Preparation of *pvdhps* nested II PCR reaction [33]

| **Reagent** | **Final conc.** | **VDHPS-NF/NR** | **VDHPS-553OF/NR** |
| --- | --- | --- | --- |
| 10x PCR Buffer | 1X | 3 µl | 3 µl |
| 50 mM MgCl2 | 3 mM | 1.8 µl |  |
|  | 1 mM |  | 0.6 µl |
| 5 mM dNTPs | 125 µM | 0.75 µl | 0.75 µl |
| 2.5 µM Primers (mix Forward & Reverse) | 250 nM | 3 µl | 3 µl |
| Taq Polymerase | 0.6 U | 0.12 µl | 0.12 µl |
| H2O |  | 21.4 µl | 22.6 µl |
| Total |  | 30 µl | 30 µl |
| Template DNA from Nested I |  | 1 µl | 1 µl |

Table S7. RFLP method for genotyping of codon 383 and 553 *pvdhps* mutations [33]

| **Codon** | **Amino Acid** | **Phenotype** | **Restriction  Enyme** | **Incubation** | **Digested size  (bp)** | **Undigested size (bp)** |
| --- | --- | --- | --- | --- | --- | --- |
| 383 | A | Wild-type | *Msp I* | 37 °C for 3 hrs. |  | 705 |
|  | G | Mutant |  |  | 655 |  |
| 553 | A | Wild-type | *Msc I* |  | 143 |  |
|  | G | Mutant |  |  |  | 171 |
